# Primary Sjögren disease patients of Asian and Middle Eastern ancestry have early‐onset but similar disease activity

**DOI:** 10.1111/imj.70075

**Published:** 2025-05-07

**Authors:** Ming Wei Lin, Sarah Volk, Adrian Y. S. Lee

**Affiliations:** ^1^ Centre for Immunology and Allergy Research, Westmead Institute for Medical Research The University of Sydney Sydney New South Wales Australia; ^2^ Department of Clinical Immunology & Allergy Westmead Hospital and ICPMR Sydney New South Wales Australia

**Keywords:** autoantibodies, autoimmunity, ethnicity, heritage, nationality, Sjögren disease

## Abstract

Sjögren disease (SjD) is a commonly encountered systemic autoimmune disease. We performed a single‐centred study of 147 patients with primary SjD and found that SjD patients of Asian and Middle Eastern ancestry had earlier onset disease than Caucasian patients. It is likely that genetic factors dictate disease characteristics and future studies to dissect the biological basis for these differences are warranted.

Sjögren disease (SjD) is one of the most common systemic autoimmune diseases typified by dryness (sicca) symptoms and arthralgias. B‐cell hyperreactivity is a common feature, and patients may have autoantibodies – particularly to Ro/La ribonucleoprotein complexes – and hypergammaglobulinaemia.^1^ SjD may overlap with other autoimmune disorders or may exist alone (primary SjD).

The causes of SjD are not clear and patients experience a heterogenous disease course with possible life‐threatening end‐organ manifestations. Hormones, biological sex, viruses and genetics are thought to be contributory to the risk and pathogenesis of this disease.^2^, ^3^ Ethnicity/race has also been implicated with differing SjD incidence rates; for example, Asian and white Caucasian females tended to have higher incidence rates (6.2–10.5 per 100 000 person‐years) over Latina females in one North American study.^4^

The Westmead Hospital Clinical Immunology clinics see an ethnically diverse range of patients due to the unique demographics of Western Sydney.^5^ As such, our SjD patients come from diverse ethnic backgrounds and present a unique opportunity to examine the impact of ancestry on disease manifestations. Because of the challenges of classing patients based on their ethnicity in a multicultural country, and because we wish to examine possible biological influences of disease, we examined the impact that ancestry (heritage) had in a single Australian centre on SjD manifestations.

This was a retrospective study of all SjD patients that were reviewed in the Clinical Immunology clinics at Westmead Hospital, Sydney, Australia. Patients reviewed in a 6‐year period from 1 June 2018 to 1 June 2024 were included and met the 2016 American College of Rheumatology/European Alliance of Associations for Rheumatology (EULAR) classification criteria for SjD.^6^ Medical records were interrogated to determine clinical and pathological features at each follow‐up timepoint. For each patient, a cumulative EULAR Sjögren syndrome disease activity index (ESSDAI) was calculated by summing the highest score for each ESSDAI domain achieved in any visit in the 6‐year period.

Patients were assigned Caucasian/European, Asian or Middle Eastern ancestry depending on their country of birth or that of their parents through direct interrogation. SPSS version 22 was used to perform statistical analyses. Ethics approval was granted by the Western Sydney Local Health District Research Office (ETH01595). All participants provided informed consent for participation.

In the 6‐year period, there was a total of 147 SjD patients with 134 (91%) females and a median age (interquartile range) of 60.7 (43.8–71.7) years by the census date. The cohort had a median follow‐up time of 4.7 (2.8–11.2) years, and there was a median of 6.0 (3.0–10.0) reviews for each patient. Five (4%) patients were lost to follow‐up during this study period. Serologically, anti‐Ro52 was found in 123 (84%), anti‐Ro60 in 120 (82%), anti‐La in 55 (37%) and rheumatoid factor in 53/138 (38%).

The ancestry groups and origins are as follows: for Caucasians (37% of total cohort), there were 37 Caucasian Australians, three Greeks, three Poles, two English, two New Zealanders and one each from Croatia, Ireland, Italy, Malta, the Netherlands, North America, Scotland and Uruguay. For the Asian group (54%), there were 26 Chinese, 22 Indians, six Filipinos, six Vietnamese, four Burmese, four Nepalese, three South Koreans, three Malaysian‐Chinese and one each from Laos, Hong Kong, Thailand, Pakistan, Indonesia and Taiwan. Finally, for the Middle Eastern group (8%), there were six Lebanese, two Egyptians, two Iraqis and one each from Jordan and Turkey.

The Asian and Middle Eastern cohorts, in general, had an earlier onset of disease than Caucasian patients (Table 1). Asian patients also display a higher degree of inflammation through greater IgG titres than Caucasian patients (Table 1). Out of the 147 SjD patients, the ESSDAIs were contemporaneously calculated for each visit by the clinician in 64 patients (44%); for the remaining patients, we retrospectively calculated the ESSDAIs from the medical records. Interestingly, the disease activity of the patients, as measured by the median ESSDAI, was similar across the heritage groups (Table 1).

**Table 1:** Sjögren disease manifestations across heritage subgroups

	Caucasian (*n* = 55)	Asian (*n* = 80)	Middle Eastern (*n* = 12)	*P*‐value
Age at symptoms (years, median)	53.0 (40.0–52.0)^a^	40.0 (31.0–52.0)^b^	40.5 (37.0–53.0)^a,b^	**0.012**
IgG (g/L, median)	12.3 (9.0–19.7)^a^	18.0 (14.2–22.5)^b^	14.0 (11.2–24.6)^a,b^	**0.001**
Median ESSDAI	2.0 (0.0–6.0)	2.5 (1.6–5.0)	1.5 (0.3–2.0)	0.079
Cumulative ESSDAI	4.0 (2.0–7.0)	4.0 (2.0–8.0)	2.0 (1.3–6.0)	0.069
ESSDAI domains				
Lymphadenopathy	5 (9)	8 (10)	1 (8)	0.974
Constitutional	4 (7)	7 (9)	1 (8)	0.953
Articular	27 (49)	39 (49)	5 (42)	0.891
Glandular	5 (9)	10 (13)	0 (0)	0.387
Cutaneous	5 (9)	11 (14)	0 (0)	0.313
Pulmonary	2 (4)	5 (6)	0 (0)	0.564
Renal	1 (2)	2 (3)	1 (8)	0.447
Peripheral nervous system	4 (7)	3 (4)	1 (8)	0.607
Central nervous system	5 (9)	4 (5)	1 (8)	0.635
Haematological	16 (29)^a^	39 (49)^b^	0 (0)^a,c^	**0.003**
Biological	24 (44)	46 (58)	7 (58)	0.260

Data in the table are presented as either median values (interquartile range) for continuous variables or *n* (%) for categorical variables. Chi‐squared or Kruskal–Wallis tests were performed as appropriate to determine differences between each subgroup. Values with superscripted letters within each row parameter differ significantly from each other (*P* < 0.05). Bolded *P*‐values represent significant *P*‐values based on an adjusted *P*‐value threshold of *P* = 0.012, controlling the false discovery rate using the Benjamini‐Hochberg method.^7^

ESSDAI, EULAR Sjögren syndrome disease activity index; IU, international units.

Next, we examined the presence of each ESSDAI domain in the 6‐year follow‐up period. There were no instances of muscular involvement, and hence, we were not able to ascertain any differences in incidence across the subgroups (data not shown). Haematological involvement (anaemia and/or cytopenias in platelets, neutrophils and/or lymphocytes) was more common in Asian than Caucasian and Middle Eastern patients, and no Middle Eastern patient had evidence of SjD‐related haematological involvement in the study period (Table 1). Examination of all these parameters for the 64 patients that contemporaneous ESSDAI calculations revealed results very similar to those of the full cohort, indicating minimal impact by retrospective calculations of ESSDAIs (data not shown).

## Discussion

This is one of the very few SjD studies that examine the impact of ancestry on SjD manifestations. In this study, we did not find evidence for greater disease activity (median and cumulative ESSDAIs) when patients were stratified according to their heritages (Table 1). The finding of earlier onset of disease in patients of Asian and Middle Eastern ancestry is less likely due to the demographics of the Western Sydney region. In the Australian 2021 census data of the local area surrounding Westmead Hospital, people of Asian and Middle Eastern ancestry tend to be of an age similar to that of people with Australian or European ancestry.^8^

One French study compared SjD patients of African versus Caucasian ancestry, finding the former group more likely to be seropositive, have greater systemic complications and higher ESSDAI,^9^ suggesting that genetic background may play an important factor in disease heterogeneity. Certainly, environmental and other biological factors could account for differences in studies across different geographical regions as well, such as vitamin D levels, viruses and microbiota.^10^ Genetics may certainly account for serological differences. The human leukocyte antigen (HLA) gene loci are among the most polymorphic among humans, and ethnic variations in frequencies of certain alleles are noted.^11^ In SjD, certain HLA‐II haplotypes, such as HLA‐DR3/DQ2, are associated with diversification of the immune response and autoantibodies to Ro/La autoantigens, which may reflect preferential T‐cell presentation of these autoantigens to antigen‐presenting cells and B cells.^12^ This genetic “hard‐wiring” means that SjD serostatuses are generally stable; yet, rare cases of new Ro/La autoantibodies emerging when serially tested may occur.^13^ Indeed, seropositivity has important prognostic implications since it helps identify a subset of SjD patients with greater extra‐glandular manifestations.^14^, ^15^

A major limitation of this study is its retrospective nature and calculation of ESSDAIs, which is highly dependent on the quality of medical records and investigations. Sixty‐six per cent of patients in this study had their ESSDAIs calculated retrospectively from medical records, which is a source of bias. All 147 patients had sufficient information to calculate ESSDAIs, due to our structured and special‐interest clinics in systemic autoimmunity at our hospital. However, we cannot exclude individual clinician and patient recall bias, which predominantly affects the clinical domains of ESSDAI. As a single‐centred study, furthermore, the patient heritage demographics would be unique to our site and may affect generalisability to the rest of Sydney, Australia or other countries. The cohort's limited representation of Middle Eastern patients (8%) may restrict the ability to identify subtle differences within this group due to the relatively small sample size. Language, cultural and socio‐economic factors may also impact on the self‐reporting of symptoms, although, in line with hospital policy, a qualified interpreter is requested for each appointment where the patient is not comfortable with English. Therefore, this study should ideally be replicated in other cohorts and geographical settings. Finally, we only assessed physician‐associated and objective features of disease, whereas patient‐centred ratings through EULAR Sjögren syndrome patient reported indices (ESSPRI), for example, would be of value to study in the future.

In summary, we find that Asian and Middle Eastern SjD patients in Western Sydney tend to have early‐onset disease, similar disease activity but, for the former, greater evidence for serological activity and cytopenias than Caucasian SjD patients. The findings of this study would be useful for prognostic and follow‐up purposes, and future studies to dissect the biological factors that contribute to disease heterogeneity would be most informative.

## Data Availability

Data are available upon reasonable request from the corresponding author.
